# Distinct effects of inflammation on preconditioning and regeneration of the adult zebrafish heart

**DOI:** 10.1098/rsob.160102

**Published:** 2016-07-20

**Authors:** Anne-Sophie de Preux Charles, Thomas Bise, Felix Baier, Jan Marro, Anna Jaźwińska

**Affiliations:** Department of Biology, Universityof Fribourg, Chemin du Musée 10, 1700 Fribourg, Switzerland

**Keywords:** thoracotomy, cryoinjury, cardiac muscle, cardiomyocyte, non-mammalian animal model, leucocytes

## Abstract

The adult heart is able to activate cardioprotective programmes and modifies its architecture in response to physiological or pathological changes. While mammalian cardiac remodelling often involves hypertrophic expansion, the adult zebrafish heart exploits hyperplastic growth. This capacity depends on the responsiveness of zebrafish cardiomyocytes to mitogenic signals throughout their entire life. Here, we have examined the role of inflammation on the stimulation of cell cycle activity in the context of heart preconditioning and regeneration. We used thoracotomy as a cardiac preconditioning model and cryoinjury as a model of cardiac infarction in the adult zebrafish. First, we performed a spatio-temporal characterization of leucocytes and cycling cardiac cells after thoracotomy. This analysis revealed a concomitance between the infiltration of inflammatory cells and the stimulation of the mitotic activity. However, decreasing the immune response using clodronate liposome injection, PLX3397 treatment or anti-inflammatory drugs surprisingly had no effect on the re-entry of cardiac cells into the cell cycle. In contrast, reducing inflammation using the same strategies after cryoinjury strongly impaired cardiac cell mitotic activity and the regenerative process. Taken together, our results show that, while the immune response is not necessary to induce cell-cycle activity in intact preconditioned hearts, inflammation is required for the regeneration of injured hearts in zebrafish.

## 1. Introduction

Preconditioning refers to the induction of cellular survival and pro-regenerative programmes by brief exposure to non-lethal noxious stimuli, which subsequently increase the resistance of tissues to further harmful injuries. Our laboratory has recently developed two independent models of cardiac preconditioning in the adult zebrafish. In the first model, we used an incision through the chest (thoracotomy) as a preconditioning stimulus. Our second model used a more remote stimulus. In this case, cardioprotection is elicited by the intraperitoneal injection of immunogenic particles [1]. In contrast to mammalian models of cardiac preconditioning, thoracotomy or injection of immunogenic particles not only invoked the expression of cardioprotective genes but also increased the mitotic activity in the adult ventricle, leading to a long-term architectural modification of the ventricle, illustrated by a thickening of the compact myocardium wall [1]. Similar to mammals, preconditioning of the adult zebrafish heart enhances regeneration. When fish are subjected to preconditioning within the week preceding the heart injury, the re-entry of cardiomyocytes (CMs) into the cell cycle is more efficient; CM dedifferentiation and cell survival are promoted [1].

Although preconditioning has proved to be remarkably reproducible in human ischaemic disorders, the underlying triggering mechanisms are still unclear [2,3]. The current hypothesis suggests that both neural and humoral aspects are at stake to elicit this phenomenon. The humoral hypothesis is supported by studies performed in rabbits or rats, where the protected phenotype could be transferred from preconditioned to naive individuals by blood transfusion [4,5]. Several effector molecules, such as bradykinin, hypoxia-inducible factor 1α (HIF-1α) or stromal-derived factor 1α (SDF-1α), have been identified by proteomic approaches; however, their use as a preconditioning factor did not give convincing results [6]. An explanation for these disappointing outcomes might come from the multifactorial nature of preconditioning [2,6,7]. For that reason, studying an upstream stimulus acting at a systemic level, such as inflammation, could be of great interest to reach a better understanding of the mechanisms underlying preconditioning.

In contrast to mammals, zebrafish are able to efficiently repair their damaged myocardium. Fibrosis is only transient, and the injured cardiac muscle is fully reconstituted by new CMs within 30–60 days [8,9]. This spectacular regenerative capacity depends on the remarkable responsiveness of zebrafish CMs to mitogenic signals throughout their adult life [8,10]. Even though many local paracrine factors promoting CM proliferation after heart injury have already been described [11–16], the role of systemic factors such as innervation or inflammation on zebrafish CM proliferation has only recently been addressed [17–19].

The innate immune system, which comprises neutrophils and macrophages, is the first defence against harmful factors and rapidly responds to signals released from damaged tissues. According to current evidence, preconditioning stimuli can activate resident phagocytic cells and cause the release of pro-inflammatory cytokines to induce the pro-survival programmes [20,21]. Nevertheless, the role of the immune response on heart preconditioning is still under debate. Contradictory results have been obtained so far, showing both a pro- and an anti-inflammatory effect of remote cardiac conditioning [22]. The exact role of inflammation on the induction of a protected phenotype in the heart needs therefore to be further investigated. Interestingly, the function of the immune response is also under debate in the context of cardiac injury. Decreasing inflammation has led to both beneficial and damaging effects on cardiac repair programmes [23,24].

In this study, we proposed to examine the role of inflammation in both heart preconditioning and regeneration. In the first part of the study, we used a thoracotomy model to investigate the mechanisms of heart preconditioning after a systemic challenge without any physical damage to the myocardium. In contrast to mammals, the systemic reaction set in motion after preconditioning in the zebrafish promoted the re-entry of cardiac cells into the cell cycle and led to long-term morphological modifications of the myocardium [1]. To address the involvement of inflammation in this phenomenon, we first characterized the spatio-temporal dynamics of leucocytes and mitotic cardiac cells after thoracotomy. We observed a correlation between the CM cell-cycle activity and the presence of leucocytes in preconditioned hearts. Unexpectedly, decreasing the immune response after thoracotomy by means of several distinct approaches had no effect on the re-entry of cardiac cells into the cell cycle, indicating that leucocytes do not act directly to promote the mitotic activity observed in preconditioned intact hearts. In the second part, we used cryoinjury as a model of cardiac infarction to assess the role of inflammation in cardiac regeneration. In regenerating hearts, we observed a similar correlation between the presence of leucocytes and cell-cycle activity to that after cardiac preconditioning. However, the analysis of cryoinjured hearts revealed an important detrimental impact of a decreased immune response on both cell mitotic activity and repair of the cryoinjured area, suggesting that immune cells are in this context a prerequisite for an efficient regeneration.

## 2. Material and methods

### 2.1. Animal procedures

This work was performed with fully grown adult fish at the age of 12–24 months. Wild-type fish were AB (Oregon), transgenic fish lines were *cmlc2:DsRed2-nuc, CD41:EGFP* and *mpeg1:EGFP* [25–27]. Before every procedure, fish were anaesthetized in 0.1% tricaine (Sigma-Aldrich). Chest wound injuries were performed as described previously [9,28]. Briefly, fish were placed ventral side up on a damp sponge, and a small incision was made through the thorax skin with iridectomy scissors. Cryoinjuries were performed as described previously [9,28]. The phagocyte population was depleted by intraperitoneal injection of 5 µl suspension of clodronate- (5 mg ml^−1^) or PBS-containing liposomes (Encapsula NanoSciences LLC) 24 h before the thoracotomy or cryoinjury procedure. The injection was repeated at 4 days post-thoracotomy (dpt) or at 4 days post-cryoinjury (dpci) to ensure a constant low level of phagocytes in clodrosome-injected fish. The inflammatory response was blocked by immersing fish in 0.05% DMSO, 500 nM PLX3397 (Plexxikon), 5 µM flumethasone (Santa Cruz Biotechnologies) or 10 µM pranlukast (Sigma-Aldrich). For all drug treatments, fish were immersed 3 days before the thoracotomy or the cryoinjury and maintained under those conditions for the following 7 days. During all treatments, fish were fed, and solutions were changed every third day. For L-plastin staining, 5 µl of 2% paraformaldehyde was injected into the thorax of euthanized animals 15 min before heart collection. This prefixation was performed to avoid corruption of the results by immune cells that are carried in the blood and that adhere to the surface of the ventricle just at the moment of heart collection. The cantonal veterinary office of Fribourg approved this experimental research on animals.

### 2.2. Immunohistochemistry and histology

At the end of each experiment, the hearts were collected and fixed overnight at 4°C in 2% paraformaldehyde. They were then rinsed in PBS and equilibrated in 30% sucrose before embedding in tissue-Tek OCT compound (Sakura Finetek Europe B.V.) and cryosectioned at a thickness of 16 µm. The immunohistochemistry procedures were performed as previously described [9]. The following primary antibodies were used: chicken anti-L-plastin at 1 : 500 (kindly provided by P. Martin, Bristol) [29], rabbit anti-MCM5 at 1 : 5000 (kindly provided by Soojin Ryu, Heidelberg), rabbit anti-DsRed (Clonetech, 632496) at 1 : 200 and mouse anti-tropomyosin (developed by Jim Jung-Ching Lin and obtained from the Developmental Studies Hybridoma Bank, University of Iowa). The Alexa-Fluor-conjugated secondary antibodies (Jackson Immunoresearch) were used at 1 : 500, and DAPI was used at 1 : 2000. Aniline blue, acid Fuchsin and Orange G (AFOG) staining were performed as previously described [16].

### 2.3. Image analysis and quantification

After antibody staining, cardiac tissue imaging was performed at 20× magnification with confocal microscopes (Leica TCS-SP5 and Leica TCS-SPE-II). At least three different pictures were taken for each heart. *n* represents the number of fish used in the experiment. ImageJ software was used to perform the subsequent image analysis. The number of proliferating non-CM nuclei was obtained by subtracting the number of *cmlc2:DsRed2-nuc*-positive nuclei from the total number of nuclei. To quantify the number of proliferating nuclei, the images of cell cycle marker (MCM5) were superimposed with the images of nuclei markers (DAPI; *cmlc2:DsRed2-nuc*). L-plastin-positive area was normalized to the total area of the ventricle. All results are expressed as the mean ± standard error of the mean. Unless specified, *p*-values were obtained by performing *t*-tests. Regeneration scores were assigned to 30 dpci regenerating hearts based on AFOG histological staining, which differentiates between the myocardium, fibrin-rich tissue and collagen fibres [17]. The scores follow the following criteria: complete regeneration = absence or residual collagen fibres; partial regeneration = advanced regeneration with persisting collagen fibres; blocked regeneration = large injured area containing provisional fibrotic matrix. Statistical significance was obtained by Fisher's exact test.

### 2.4. Protein homology analysis

To test if the binding pockets of c-Kit and c-FMS proteins are conserved through vertebrates, using T-Coffee [30] we aligned the sequences of *Homo sapiens* c-FMS (NP_005202.2) and c-Kit (NP_000213.1) with their *Danio rerio* orthologues c-FMS (NP_571747.1), c-Kita (NP_571128.1) and c-Kitb (NP_001137390.1).

## 3. Results

### 3.1. Cardiomyocyte proliferation correlates with the presence of leucocytes after thoracotomy

We recently established thoracotomy as a model of heart preconditioning in the adult zebrafish [1]. While leaving the heart untouched, the thoracotomy procedure exposes the cardiac structures to many aggressions, such as mechanical and osmotic stress or bacterial contamination, triggering an inflammatory response. To test whether inflammation might cause the increased CM mitotic activity seen after thoracic incision, we decided to characterize the distribution of leucocytes after thoracotomy. For this, we used an antibody against L-plastin, a leucocyte-specific actin-bundling protein [31]. To validate L-plastin as a suitable leucocyte marker, transgenic reporter lines for specific haematopoietic lineages were used: *mpeg1:EGFP* fish were used to visualize macrophages, whereas *CD41:EGPF* fish were selected to identify thrombocytes [26,27]. Remarkably, all *mpeg1:EGFP*-positive cells were L-plastin-positive (electronic supplementary material, figure S1*a*). At 7 dpt, *mpeg1:EGFP*-positive cells accounted for approximately 10% of L-plastin-positive cells (data not shown). In contrast, no overlap was found between L-plastin staining and the thrombocytes marker *CD41:EGFP* (electronic supplementary material, figure S1*b*). Moreover, no L-plastin was detected in the blood clots occasionally trapped in the lumen of the heart (electronic supplementary material, figure S1*c*). In summary, these co-stainings showed that L-plastin does not label thrombocytes or erythrocytes, but colocalizes with the specific leucocyte transgenic reporter, *mpeg1*. We concluded that L-plastin is a valid leucocyte marker.

**Figure 1.**
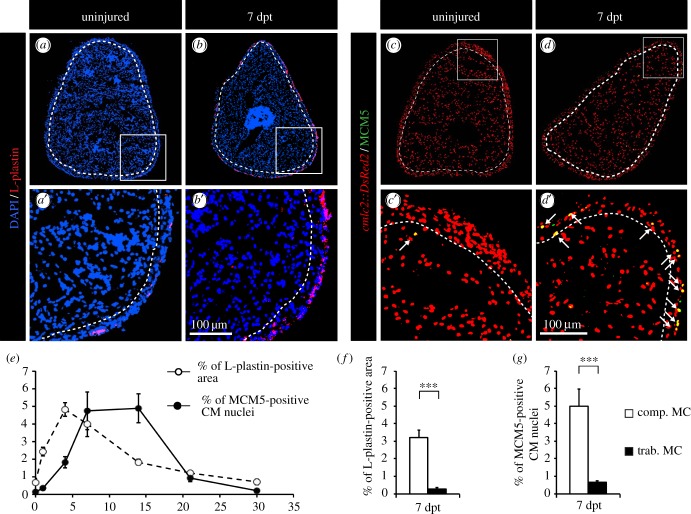
CM proliferation is correlated with the distribution of phagocytic immune cells. (*a*,*b*) Representative sections of the hearts reveal a few L-plastin-positive cells in uninjured fish in comparison to fish at 7 days post-thoracotomy (dpt) with the abundant L-plastin staining in the compact myocardium. A white dashed line separates compact (comp. MC) and trabecular (trab. MC) myocardium. (*c*,*d*) Representative sections of the heart of *cmlc:DsRed2-nuc* transgenic fish (red, marker of CM nuclei) labelled with the G1/S-phase marker MCM5 (green) reveal an increased mitotic activity at 7 dpt. Arrows indicate double-positive cells. (*e*) Quantification of the L-plastin-positive area (white circles) and of the number of proliferative CMs (black circles) in sections of uninjured hearts, and at 1, 4, 7, 14, 21 and 30 dpt. (*f*,*g*) Quantification of L-plastin-labelled leucocytes and MCM5-positive CMs in the two distinct myocardium compartments reveals a spatial correlation of both distributions at 7 dpt. (*n* ≥ 4 hearts; ≥2 sections per heart; ***p < 0.001).

To detect any correlation between mitotic activity and inflammation, we performed a spatio-temporal description of the distribution of leucocytes and cycling CMs after thoracotomy (figure 1). The presence of leucocytes was increased rapidly after thoracotomy to peak at 4 dpt. The inflammatory response then slowly decreased to reach its normal level by 30 dpt (figure 1*e*). CM cell-cycle activity was assessed by counting the number of MCM5-positive CMs. Remarkably, after thoracic incision, CM mitotic activity slowly increased to reach a plateau between 7 and 14 dpt with approximately 5% of cycling CMs, and finally decreased to reach its normal level at 30 dpt (figure 1*e*).

The zebrafish ventricle is composed of two distinct compartments: a dense cortical layer (compact myocardium) that surrounds a spongy inner structure (trabecular myocardium) [32]. We have previously described that most cycling CMs are located in the compact myocardium after preconditioning. Based on this observation, we asked whether leucocytes were evenly scattered through both myocardial compartments. Strikingly, we observed that leucocytes accumulated in the compact myocardium (figure 1*a*,*b*,*f*), where cycling CMs were observed (figure 1*c*,*d*,*g*). The concomitance of the inflammatory response and of the entry of CMs into the cell cycle suggests a correlation between these two processes.

### 3.2. Leucocyte accumulation is not required to stimulate cardiomyocyte proliferation after thoracotomy

**Figure 2.**
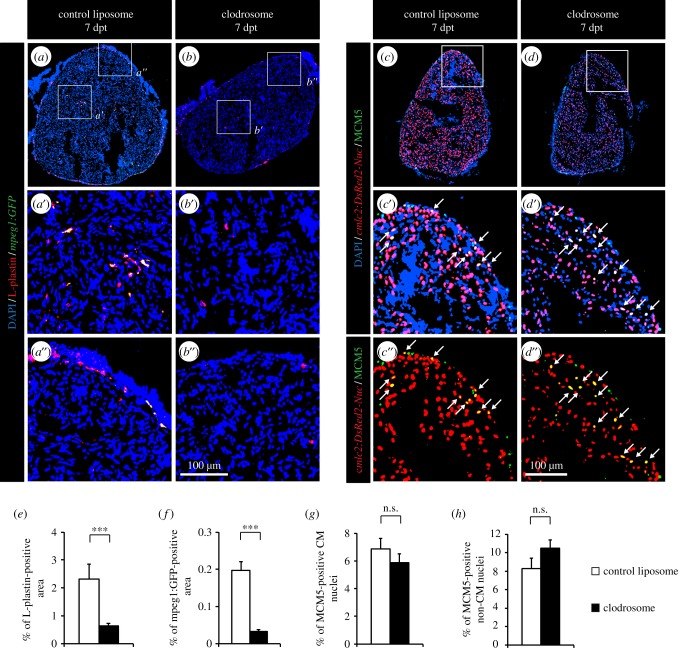
Depleting phagocytes by injection of clodronate liposomes does not affect CM proliferation. (*a*,*b*) The impact of control and clodronate liposome injection on the presence of leucocytes was tested by labelling the hearts of *mpeg1:GFP* transgenic fish (green) with L-plastin (red). (*c*,*d*) CM proliferation was quantified by measuring the number of CMs co-expressing DsRed2-nuc (red) and MCM5 (green). Double-positive cells are indicated with arrows. (*e*,*f*) Quantification of L-plastin- and *mpeg1:GFP*-positive area. (*g*,*h*) Quantification of proliferative CM and non-CM nuclei. (*n* ≥ 4 hearts; ≥2 sections per heart; ***p < 0.001; n.s., non-significant).

To test the role of the leucocytes in the initiation of the mitotic response induced in our cardiac preconditioning model, we applied diverse experimental approaches to modulate the immune response. First, the overall phagocyte population was depleted by an intraperitoneal injection of clodrosomes, which are cytotoxic clodronate encapsulated liposomes, one day before the thoracotomy and at 4 dpt. In the mouse heart, this method specifically reduces the cardiac mononuclear phagocyte population without altering neutrophils [23]. We found that clodrosome-injected fish displayed a fourfold reduction of L-plastin-positive area in the heart at 7 dpt, compared with fish injected with control liposomes (figure 2*a*,*b*,*e*). Similarly, very few *mpeg1:EGFP*-positive cells could be detected after clodrosome injections (figure 2*a*,*b*,*f*). These results support clodrosome injection as a valid method to deplete phagocytes in the zebrafish. Surprisingly, this decreased immune response had no effect on the cell-cycle activity of CM or non-CM nuclei (figure 2*c*,*d*,*g,h*).

**Figure 3.**
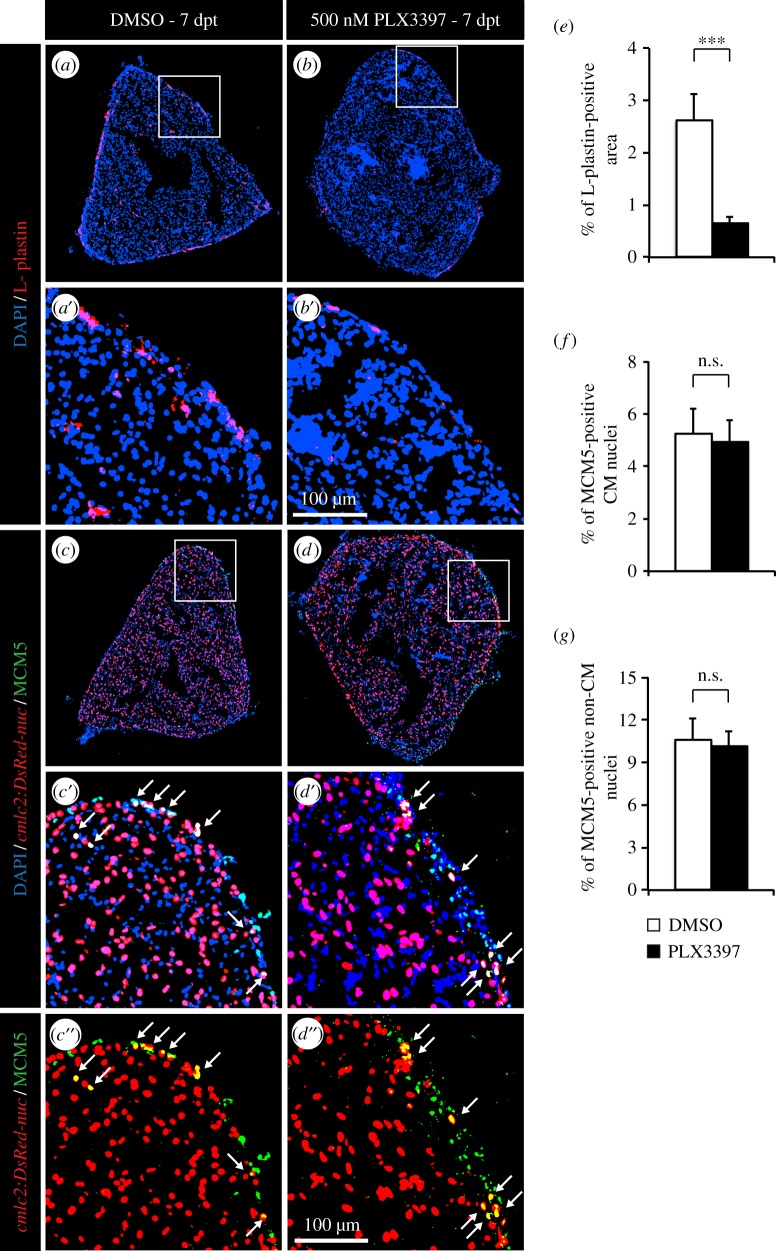
Depletion of leucocytes by PLX3397 treatment does not affect CM proliferation. (*a*,*b*) The impact of PLX3397 treatment on the presence of leucocytes was tested by labelling the hearts with L-plastin (red). (*c*,*d*) CM proliferation was quantified by measuring the number of CMs co-expressing *cmlc:DsRed2-nuc* (red) and MCM5 (green). Arrows indicate double-positive cells. (*e*) Quantification of L-plastin-positive area at 7 dpt. (*f*,*g*) Quantification of proliferative CM and non-CM nuclei at 7 dpt. (*n* ≥ 4 hearts; ≥2 sections per heart; ***p < 0.001; n.s. non-significant).

In a second approach, the immune response after thoracotomy was modulated using PLX3397, a selective inhibitor of c-Fms/CSF1R and c-Kit tyrosine kinases. These receptors regulate the proliferation of multipotent haematopoietic progenitor cells and their myeloid differentiation [33]. In mammals, PLX3397 has been shown to inhibit macrophage migration and proliferation and to decrease the production of pro-inflammatory cytokines [34]. To verify if PLX3397 might be an efficient c-Fms/CSF1R and c-Kit inhibitor in zebrafish, we aligned the *H. sapien*s and *Danio rerio* c-Fms and c-Kit orthologue proteins (electronic supplementary material, figure S2). We focused this comparison on the region that interacts with PLX3397 (*H. sapiens* c-Kit L521–L865; *H. sapiens* c-Fms L514–K870) [35]. Both proteins were well conserved between these two species, and all residues making direct contact with the inhibitor were preserved, suggesting that PLX3397 might work as a specific c-Fms/CSF1R and c-Kit inhibitor also in zebrafish. Accordingly, PLX3397-treated fish displayed a threefold decrease of the presence of leucocytes in the heart at 7 dpt (figure 3*a*,*b,e*). Despite this, CM cell-cycle activity was not affected (figure 3*c*,*d*,*f*,*g*).

**Figure 4.**
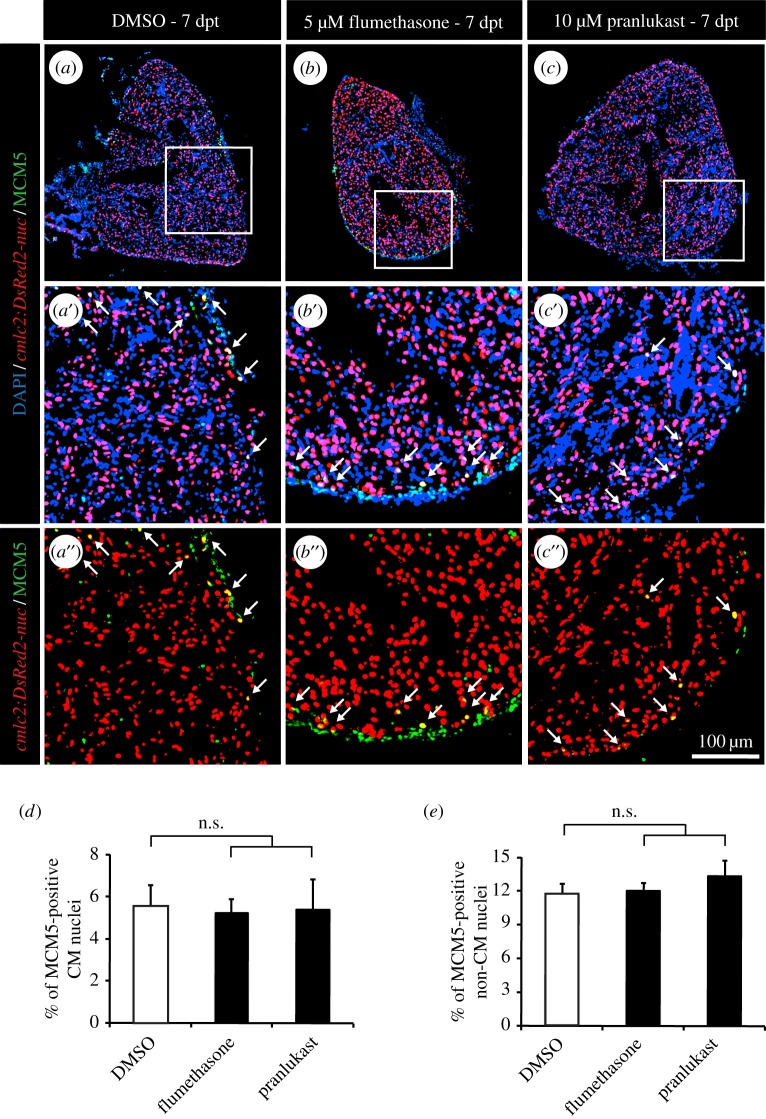
Wide anti-inflammatory drugs do not affect CM proliferation after thoracotomy. (*a*–*c*) Representative heart images of *cmlc2:DsRed-nuc* fish (red) treated with 0.05% DMSO (*a*), 5 µM flumethasone (*b*) or 2.5 µM pranlukast (*c*) labelled with the cell cycle marker MCM5 (green) at 7 dpt. Arrows indicate double-positive cells. (*d*,*e*) Quantification of MCM5-positive CM and non-CM nuclei at 7 dpt. (*n* ≥ 4 hearts; ≥2 sections per heart; n.s., non-significant).

Because both methods, clodronate liposomes and PLX3397, mainly affect the macrophage lineage, widely acting anti-inflammatory drugs were used to exclude any role of other immune mechanisms in the induction of CM proliferation (figure 4). Even so, reducing the immune response using flumethasone (figure 4*b*) or pranlukast (figure 4*c*) did not affect the rate of re-entry into the cell cycle (figure 4*d*,*e*).

Taken together, we concluded that cardiac preconditioning is accompanied by an accumulation of leucocytes in the uninjured myocardium. However, the presence and function of these immune cells are not essential for triggering cell-cycle progression in the intact zebrafish heart.

### 3.3. Cycling cardiomyocytes are associated with the presence of leucocytes after cryoinjury

**Figure 5.**
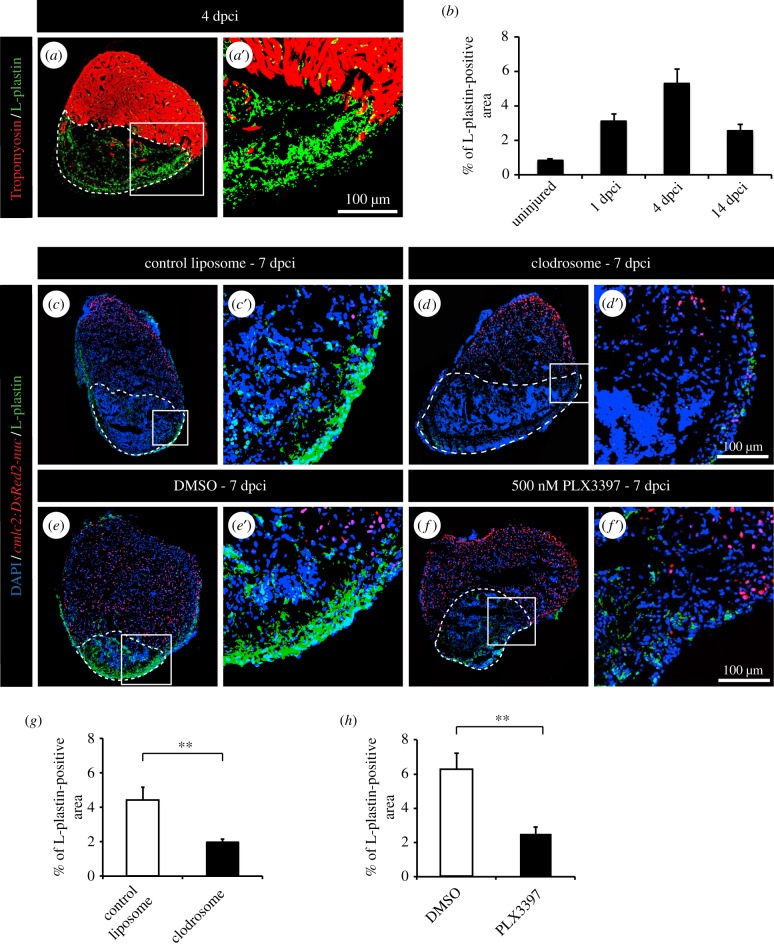
Clodronate liposome injections and PLX3397 treatment efficiently deplete phagocyte populations after cryoinjury. (*a*) Representative image of cryoinjured heart at 4 days post-cryoinjury (dpci) labelled with antibody against tropomyosin (red) to demarcate the remaining myocardium and L-plastin (green) to reveal leucocytes. The cryoinjured area is identified by the absence of tropomyosin (encircled by dashed line). (*b*) Quantification of the L-plastin-positive area in sections of uninjured hearts, and at 1, 4 and 14 dpci. (*c*–*f*) Representative images of hearts of transgenic fish *cmlc2:DsRed2-nuc* at 7 dpci with L-plastin staining (green). The cryoinjured area is identify by the absence of *cmlc2:DsRed2-nuc* expression (encircled by dashed line). (*c*,*d*) Hearts after control and clodronate liposome (clodrosome) injections. (*e*,*f*) Hearts after 0.05% DMSO or 500 nM PLX3397 treatment. (*g*,*h*) Quantification of L-plastin-positive area after clodronate liposome injection (*g*) and PLX3397 treatment (*h*) at 7 dpci. (*n* ≥ 4 hearts; ≥2 sections per heart; **p < 0.01).

Although inflammation is associated but not required for heart conditioning, we decided to address the role of leucocytes during heart regeneration. To induce myocardial injury, we applied the cryoinjury model, in which approximately 20% of the ventricle is damaged by exposure to a precooled metal probe [28]. Within the first week after the procedure, the damaged part of the myocardium becomes replaced with provisional fibrotic tissue. Within four weeks, this fibrotic tissue undergoes remodelling to repair the injured ventricle and progressively withdraws to give space for the regenerating myocardium [36]. After cryoinjury, CM mitotic activity slowly increased to reach a plateau between 7 and 21 dpci with approximately 10% of proliferating CMs, and finally decreased to reach its normal level at 60 dpci [9]. The complete restoration of the cardiac tissue is achieved in four to eight weeks [9,37]. To determine the involvement of immune response in heart regeneration, we analysed the distribution of L-plastin-expressing cells at different regenerative phases. Shortly after cryoinjury, at 1 and 4 dpci, leucocytes infiltrated the myocardium and abundantly accumulated in the post-injury zone, consistent with the stimulation of immune response in the damaged tissue (figure 5*a*,*b*). At 14 dpci, the number of L-plastin-positive cells declined as regeneration progressed (figure 5*b*).

### 3.4. Leucocytes promote the re-entry of cardiomyocytes into the cell cycle after cryoinjury

**Figure 6.**
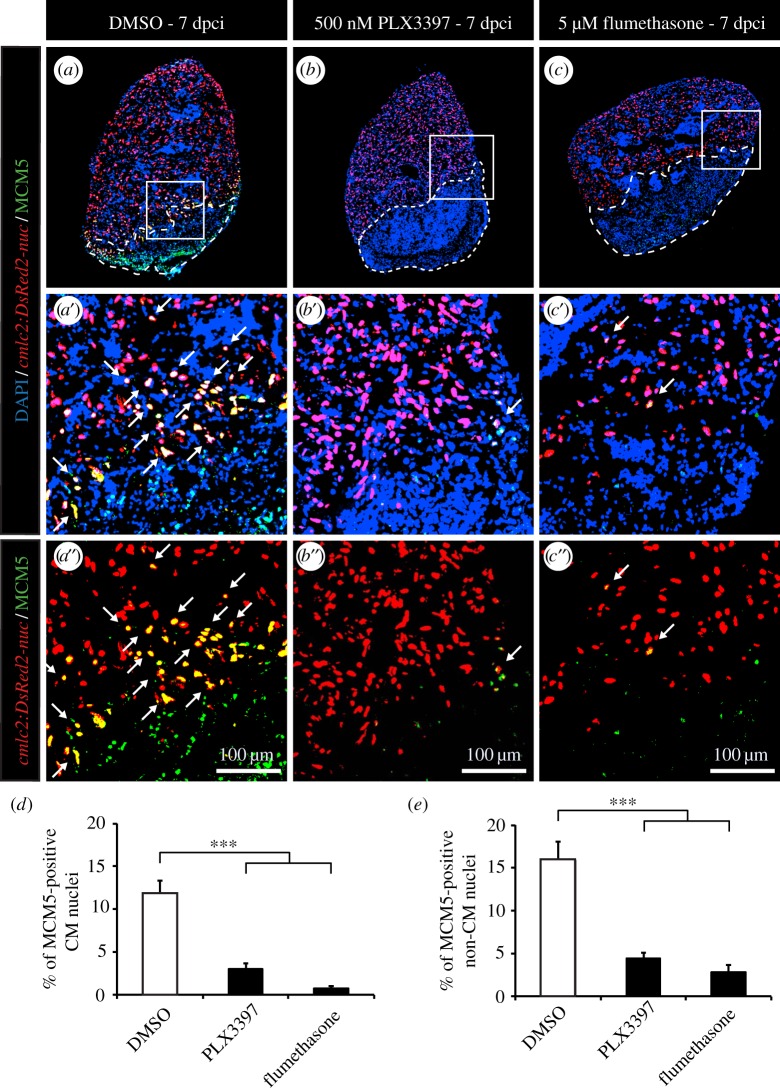
Inhibition of inflammation strongly decreases CM proliferation after cryoinjury. (*a*–*c*) Representative images of the hearts of *cmlc2:DsRed-nuc* fish (red) treated with 0.05% DMSO (*a*), 500 nM PLX3397 (*b*) or 5 µM flumethasone (*c*) labelled with the cell cycle marker MCM5 (green). The cryoinjured area is detected by the absence of *cmlc2:DsRed-nuc* (encircled with a dashed line). Arrows indicate double-positive cells. (*d*,*e*) Quantification of MCM5-positive CMs and non-CMs at 7 dpci. (*n* ≥ 4 hearts; ≥2 sections per heart; ***p < 0.001).

**Figure 7.**
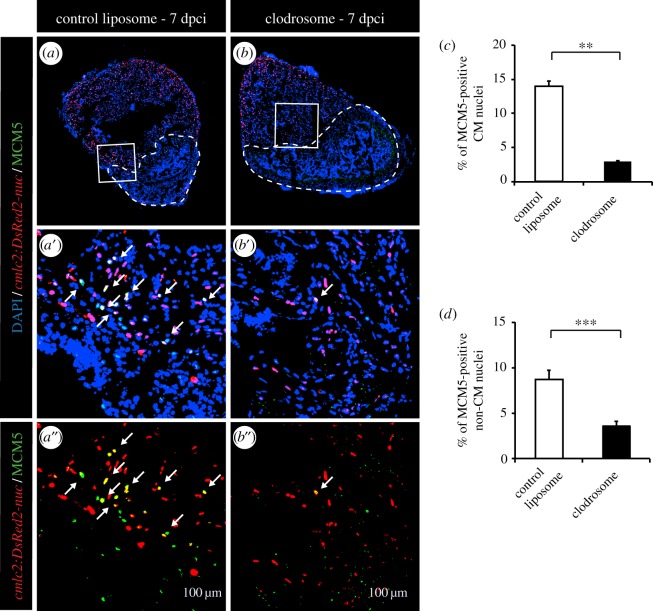
Phagocyte depletion strongly decreases CM proliferation after cryoinjury. (*a*,*b*) Representative images of the hearts of *cmlc2:DsRed-nuc* fish (red) injected with control liposomes (*a*) or injected with clodronate liposomes (*b*) labelled with the cell cycle marker MCM5 (green). The cryoinjured area is detected by the absence of *cmlc2:DsRed-nuc* (encircled with a dashed line). Arrows indicate double-positive cells. (*c*,*d*) Quantification of MCM5-positive CMs and non-CMs at 7 dpci. (*n* ≥ 4 hearts; ≥2 sections per heart; **p < 0.01; ***p < 0.001).

To assess the role of inflammation on CM cell-cycle activity after ventricular cryoinjury, we applied the same experimental strategies as after thoracotomy. Again, we were able to efficiently reduce the phagocyte population with PLX3397 treatment or intraperitoneal injection of clodrosomes, as illustrated by L-plastin staining (figure 5*c*–*f*). With both strategies, more than a twofold decrease of leucocytes was observed in the hearts at 7 days after cryoinjury (figure 5*g*,*h*). In this context, however, the reduced immune response strongly suppressed the stimulation of the mitotic activity (figure 6*a*,*b*,*d*,*e* and figure 7). This effect was observed for both CM and non-CM nuclei (figures 6*d*,*e* and 7*c*,*d*). Likewise, treating fish with the wide anti-inflammatory drug flumethasone severely impaired the re-entry of cardiac cells into the cell cycle at 7 dpci (figure 6*c*,*d*,*e*).

### 3.5. The deposition of a transient scar is dependent on leucocytes after cryoinjury

**Figure 8.**
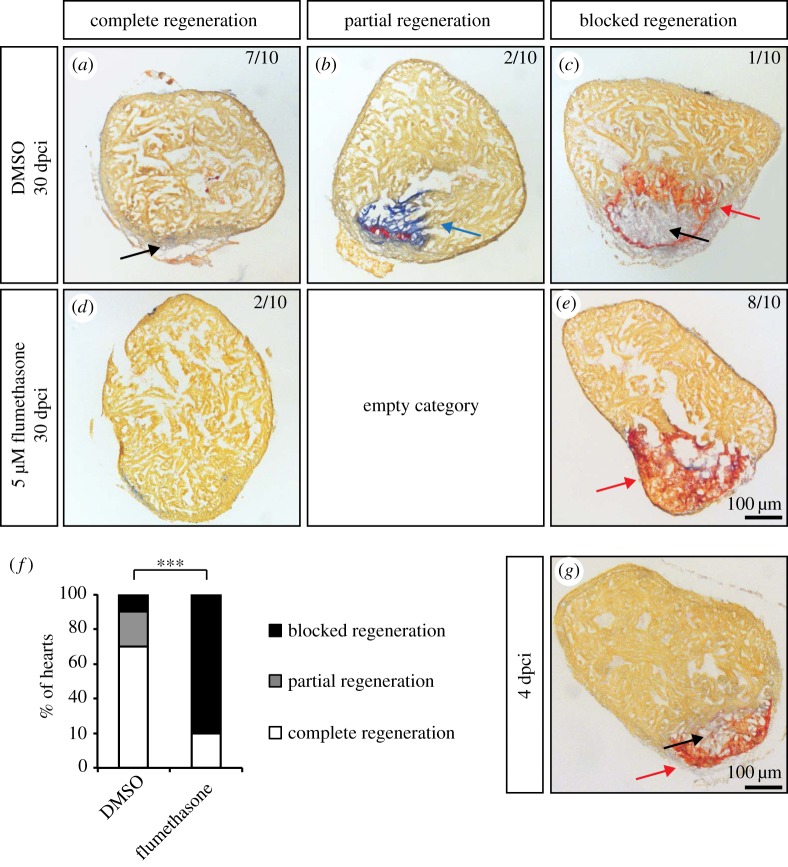
Flumethasone treatment inhibits matrix remodelling after cryoinjury and heart regeneration. (*a*–*e*) Representative sections of cryoinjured hearts at 30 dpci labelled with AFOG staining, which detects collagen in blue (blue arrow), fibrin-like matrix in red (red arrows) and muscle in orange, after 30 days of treatment with 0.05% DMSO and 5 µM flumethasone. The newly formed myocardium is indicated by black arrows. (*f*) Quantification of heart regeneration according to the criteria described in Materials and methods. (*g*) Representative example of a heart at 4 dpci labelled with AFOG staining. The cryoinjured zone in the heart at 4 dpci contains a provisional fibrotic matrix (red), which displays a similarity to the matrix of flumethasone-treated hearts at 30 dpci. (*n* ≥ 10; ***p < 0.001, Fisher's exact test).

Moreover, fish treated with flumethasone for one month after cryoinjury clearly exhibited a blocked regenerative process, as revealed by histological staining (figure 8*a*–*e*). In the majority of control fish, regeneration was almost completed (figure 8*d*–*f*). At this stage, fibrin deposits were completely resolved, and only small size scars retaining collagen fibres were observed (figure 8*a*–*c*). In contrast, the flumethasone-treated fish displayed blocked regeneration (figure 8*d*,*e*,*f*), which was reminiscent of the early regenerative phase, such as 4 dpci (figure 8*g*). Fibrin-like material persisted in the cryoinjured areas, and the deposition of a collagen network necessary to support the migration of the newly formed cardiac cells was severely impaired (figure 8*e*). To exclude any side effect of the flumethasone treatment, we decided to validate our observations using the clodronate liposome approach. For this, fish were injected with control or clodronate liposomes 24 h before the cryoinjury procedure. Liposome injections were then repeated every 4 days for 2 weeks. Again, an impairment of regeneration was found in fish in which the immune response was blocked by clodronate liposomes (data not shown), confirming a specific role of leucocytes in the initiation of cardiac healing after injury. Taken together, our results demonstrate thus that the key regenerative processes of transient scar deposition and CM proliferation are dependent on the appropriate immune response after heart injury.

## 4. Discussion

In mammalian studies, conflicting data have been obtained so far on the role of inflammation on heart preconditioning [22]. For instance, it has been demonstrated that a brief forearm ischaemia suppresses pro-inflammatory genes expression in circulating human leucocytes [38], whereas neutrophil adhesion and phagocytosis are reduced [39]. At the same time, an increased production of tumour necrosis factor-α, interleukin-1β and -8 has been detected in patients subjected to remote ischaemic preconditioning before cardiopulmonary bypass [40]. Further studies are therefore needed to precisely define the role of inflammation during cardiac preconditioning.

To address this question in the zebrafish, we first characterized the spatio-temporal dynamics of the immune response after thoracotomy. We observed a correlation between the presence of inflammatory cells and mitotic nuclei after heart preconditioning, suggesting a link between these two processes. We used two distinct strategies that effectively depleted the macrophage population and two different immunosuppressive drugs. None of these techniques, however, had an effect on cardiac cell proliferation. Thus, leucocytes are not necessary to induce proliferation of cardiac cells in the context of heart preconditioning.

In contrast to this result, the immune response is necessary for mitotic activity, the transient scar deposition and heart regeneration after cryoinjury. These results are consistent with previously published studies. In neonatal mice, injections of liposomal clodronate robustly impair heart regeneration after myocardial infarction [23,24]. Likewise, inhibiting phagocyte recruitment with the glucocorticoid beclomethasone strongly reduces zebrafish heart regenerative capacities after ventricular resection [18]. Taken together, these observations highlight the importance of leucocytes in the context of a regenerative process, where the immune response cleans the damaged area and creates a favourable environment for tissue rebuilding. These immune cells are essential to ensure the re-entry of cardiac cells into the cell cycle and to pilot the injured heart towards an efficient regeneration.

Besides, the clear phenotype observed in the cryoinjury model confirms that the absence of effect of an altered immune system after heart preconditioning was not due to any dosage issues. We applied the same protocols and drug dosage on both models. Moreover, we achieved a better reduction of L-plastin-positive cells after preconditioning than after cryoinjury. Therefore, we can exclude the possibility that the negative results obtained with our thoracotomy model were caused by a too gentle depletion of the phagocytes.

In summary, we discovered that the function of inflammation on the mitotic activity of cardiac cells is context-dependent. In intact preconditioned heart, the entry of CM and non-CM cells into the cell cycle is unresponsive to leucocyte accumulation. These results suggest that the factors triggering the mitotic cycle of cardiac cells are not secreted by local leucocytes. In contrast, in injured hearts, phagocytic cells are key components of the regenerative process. When their function is depleted, cellular debris and fibrin are not efficiently cleared. The persisting damaged tissue is likely to impair remodelling of the post-infarcted ventricle, which directly or indirectly blocks cardiac cell proliferation and migration to achieve heart regeneration. Although organ regeneration requires the inflammatory phase, the preconditioning evokes regenerative programmes through leucocyte-independent mechanisms that remain yet uncharacterized.

## Supplementary Material

Figure S1: L-Plastin staining colocalizes with the macrophage marker mpeg1

## Supplementary Material

Figure S2: PLX3397 binding pocket is conserved between human and zebrafish.
