# Supplementary material for: Distinct effects of inflammation on preconditioning and regeneration of the adult zebrafish heart
Source: Open Biol. 2016 Jul 20;6(7):160102. doi: 10.1098/rsob.160102 (PMC4967830; doi:10.1098/rsob.160102)
Supplement: Figure S1: L-Plastin staining colocalizes with the macrophage marker mpeg1 [file rsob160102supp1.pdf]

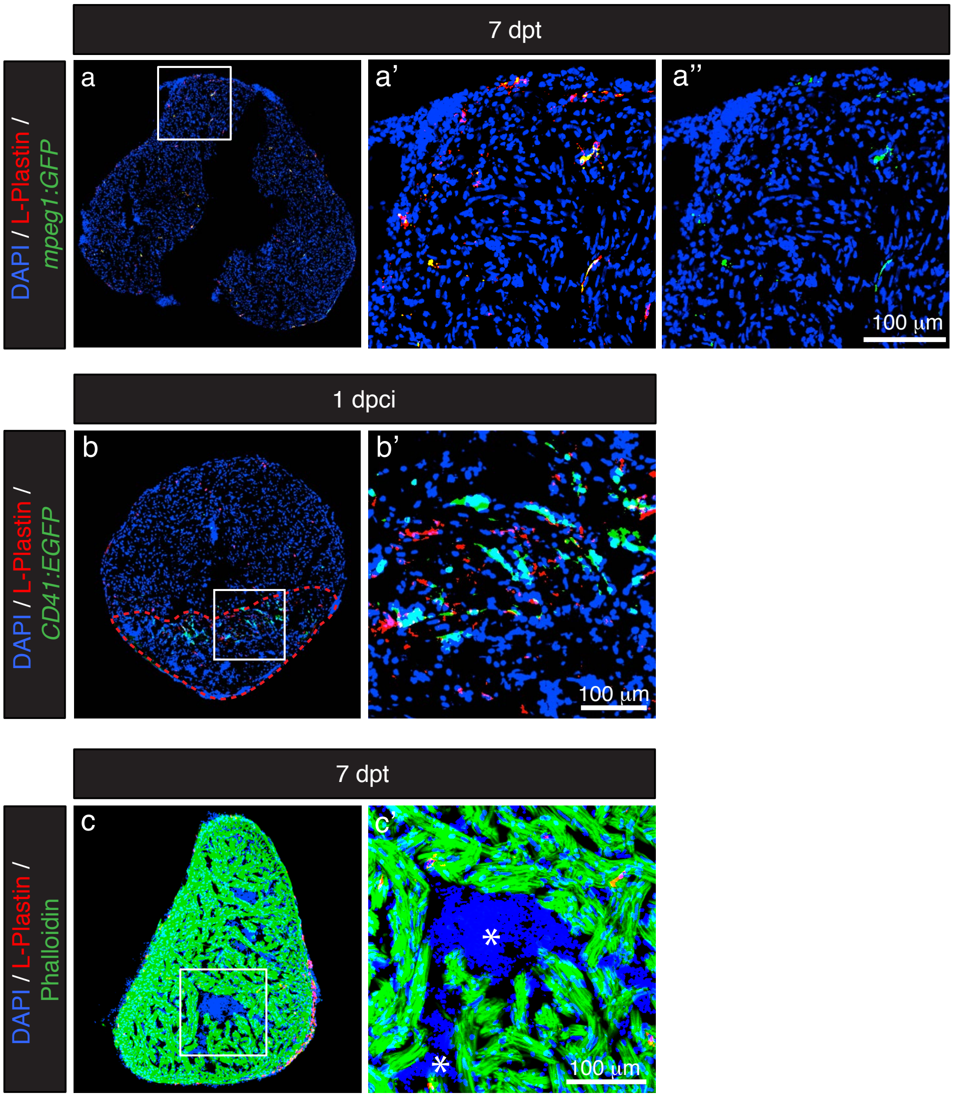

Figure S1: L-Plastin staining colocalizes with the macrophage marker mpeg1

(a) Representative section of the heart of mpeg1:GFP fish (green, marcrophage reporter line) at 7 dpt labeled with antibodies against L-Plastin (red, leukocyte protein) display an overlap of both markers. (b) Representative section of the heart of CD41:GFP fish (green, thrombocyte reporter line) at 1 dpci labeled with L-Plastin (red) display no overlap between both markers. Cryo-injured area is encircled with the red dashed line. (c) No L-Plastin (red) was detected in luminal blood (asterisk) of the heart at 7 dpt.
