# Supplementary material for: Distinct effects of inflammation on preconditioning and regeneration of the adult zebrafish heart
Source: Open Biol. 2016 Jul 20;6(7):160102. doi: 10.1098/rsob.160102 (PMC4967830; doi:10.1098/rsob.160102)
Supplement: Figure S2: PLX3397 binding pocket is conserved between human and zebrafish. [file rsob160102supp2.pdf]

a

```
Human_KIT      LFTPLLIGFVIVAGMMCIIVMILTYKYLQKPMYEVQWKVVEEINGNNYVYIDPTQLPYDH
Danio_KITA     LFTPLLIGFVAAAVILVLILIVLTYKYMQKPKYQIQWKVIEGIHGNNYVYIDPTQLPYDH
Danio_KITB     LFTPLLSGVVSTSVLLSFILVVLLYKYMQKPKYEIHWKVIDSFDGNNYTYIDPTQLPYDP
               ****** *.*  .:  ::  :*:::* ***:*** *:::***::  :.****.**********.
                    Transmembrane region          Juxtamembrane domain

Human_KIT      KWEFPRNRLSFGKTLGAGAFGKVVEATAYGLIKSDAAMTVAVKMLKPSAHLTEREALMSE
Danio_KITA     QWEFPRDKLRFGKTLGSGAFGKVVEATAYGMSKADTVMTVAVKMLKPSAHATEKEALMSE
Danio_KITB     KWEFPRERLRFGKILGSGAFGKVVAATAYGLCSADTVTTVAVKMLKPSAHSTEKEALMSE
               :*****::* *** **:******* *****: .:*:. ************ **:******
                                  P-loop

Human_KIT      LKVLSYLGNHMNIVNLLGACTIGGPTLVITEYCCYGDLLNFLRRKRDSFICSKQEDHAEA
Danio_KITA     LKVLSYLGNHINIVNLLGACTVGGPTLVITEYCCFGDLLNFLRRRRVYFYYTTLG---ED
Danio_KITB     LKVLSYIGNHINIVNLLGACTVGGPTLVITEYCCYGDLLNFLRRKRDAFFSSKTG----D
               ******:***:**********:************:*********:*   *   :.

Human_KIT      ALYKNLLHSKESSCSDSTNEYMDMKPGVSYVVPTKADKRRSVRIG-SYIERDVTPAIMED
Danio_KITA     AYYRNVMMQSEP--NDSRNGYMTMKPSVLGIL--SSENRRSLNKGDSYSDSDAVSEILQE
Danio_KITB     GYYKNLLSQTQPSIEGTDNGYMPMR---------------------SYQKRSNQTEWCDD
               . *:*::  ..:.   ..: * ** *:                   ** . .   .   ::
                    Kinase insertion domain

Human_KIT      -DELALDLEDLLSFSYQVAKGMAFLASKNCIHRDLAARNILLTHGRITKICDFGLARDIK
Danio_KITA     -DGLTLDTEDLLSFSYQVAKGMDFLASKNCIHRDLAARNILLTQGRVAKICDFGLARDIT
Danio_KITB     KDDLSLDTEDLLSFSYQVAKGMDFLTSKNCIHRDLAARNVLLTQGRVAKICDFGLARDIT
                *  *:** ************** **:*************:***:**::***********.
                                                 Catalytic loop       Activation

Human_KIT      NDSNYVVKGNARLPVKWMAPESIFNCVYTFESDVWSYGIFLWELFSL
Danio_KITA     TDSNYVVKGNARLPVKWMSPESIFECVYTFESDVWSYGILLWEIFSL
Danio_KITB     RDSNYVLRGNARLPVKWMSPESLFACVYTFESDVWSYGILLWEIFSL
                *****::**********:***:* **************:***:***
                 loop
```

b

```
Human_FMS      LFTPVVVACMSIMALLLLLLLLLYKYKQKPKYQVRWKIIESYEGNSYTFIDPTQLPYNE
Danio_FMS      IFTSAMCGSTVAMVVLGLLLIFMIYKYKQKPRYEIRWKIIEATNGNNYTFIDPTQLPYNE
               :**..:  ..     *.:* ***::::*******:*::******:  :**.*************
                    Transmembrane region          Juxtamembrane domain

Human_FMS      KWEFPRNNLQFGKTLGAGAFGKVVEATAFGLGKEDAVLKVAVKMLKSTAHADEKEALMSE
Danio_FMS      KWEFPRDKLKLGKTLGAGAFGKVVEATAYGLGKEDNITRVAVKMLKASAHPDEREALMSE
               ******::*::*****************:******  :  :*******::**.**:******
                                   P-loop

Human_FMS      LKIMSHLGQHENIVNLLGACTHGGPVLVITEYCCYGDLLNFLRRKAEAMLGPSLSPGQDP
Danio_FMS      LKILSHLGQHKNIVNLLGACTHGGPVLVITEYCCHGDLLNFLRSKAENFLNFVMTIPNFP
               ***:******:***********************:********* *** :*.  ::  : *

Human_FMS      EGGVDYKNIHLEKKYVRRDSGFSSQGVDTYVEMRPV-STSSNDSFSEQDLDKEDGRPLEL
Danio_FMS      EPMTDYKNVSTERMFVRSDSGISSTCSDHYLDMRPVTSRPTNSALDSSSECQEDSWPLDM
               *   .****:   *:  :** ***:**    *  *::**** *  .:*.::....   :**.  **::
                                   Kinase insertion domain

Human_FMS      RDLLHFSSQVAQGMAFLASKNCIHRDVAARNVLLTNGHVAKIGDFGLARDIMNDSNYIVK
Danio_FMS      DDLLRFSSQVAQGLDFLAAKNCIHRDVAARNVLLTNSRVAKICDFGLARDIMNDSNYVVK
                ***:*********:  ***:****************.:****  **************:**
                                   Catalytic loop       Activation loop

Human_FMS      GNARLPVKWMAPESIFDCVYTVQSDVWSYGILLWEIFSLGLNPYPGILVNSKFYKLVK
Danio_FMS      GNARLPVKWMAPESIFECVYTVQSDVWSYGIMLWEIFSLGKSPYPNILVDSKFYKMIK
               ****************:**************:********  .***.***:*****::*
```

Figure S2: PLX3397 binding pocket is conserved between human and zebrafish.

Alignment of the protein sequences of Homo sapiens KIT with Danio rerio KITA and KITB (a), and of Homo sapiens FMS with Danio rerio FMS (b) with T-Coffee. Important regions are labeled below the sequences. The residues making direct contact with the FMS/KIT inhibitor are indicated in red. "*"-asterisk denotes the residue at which the position is exactly the same. ":"-colon indicates residues at which positions are very similar, and "."-dot, residues that are more or less similar.
